# Different modes of stimulation at the Neiguan acupoint (PC6) regulate different EEG microstates under VR immersion conditions

**DOI:** 10.3389/fncom.2026.1938329

**Published:** 2026-10-22

**Authors:** Zixin Huo, Simin Zhao, Qi Liu, Shuyong Jia, Qiuyue Lyu, Xixian Liu, Na Tu, Xiaojing Song, Shuyou Wang, Weibo Zhang, Lianqi Geng, Liming Sun, Yi Guo, Guangjun Wang

**Affiliations:** 1 Institute of Acupuncture and Moxibustion, China Academy of Chinese Medical Sciences, Beijing, China; 2 Tianjin University of Traditional Chinese Medicine, Tianjin, China; 3 National Clinical Research Center for Chinese Medicine, Tianjin, China; 4 Binhai New Area Hospital of Traditional Chinese Medicine, Tianjin, China; 5 Beijing First Hospital of Integrated Chinese and Western Medicine, Beijing, China

**Keywords:** electroencephalography, mechanism, microstates, neuroscience, thermal stimulation, transcutaneous electrical acupoint stimulation, virtual reality motion sickness (VRMS)

## Abstract

**Introduction:**

Acupuncture at the Neiguan acupoint (PC6) alleviates postoperative and chemotherapy-induced nausea and vomiting. Nevertheless, the central regulatory effects of PC6 stimulation on virtual reality motion sickness (VRMS), a form of physiological vertigo triggered by visual-vestibular sensory conflict, and the corresponding alterations in electroencephalogram (EEG) microstate characteristics remain unclear. Using EEG microstate analysis as the primary approach, this study investigated the effects of different PC6 stimulation modalities on dynamic brain functional changes during VRMS, aiming to further elucidate the potential neural mechanisms underlying acupoint stimulation in VRMS.

**Methods:**

Participants were recruited based on their Motion Sickness Susceptibility Questionnaire scores, with a cut-off value of ≥34 used as the inclusion criterion. A total of 200 healthy participants were enrolled and randomly assigned to four equal groups (*n* = 50 per group): control (32 °C), 40 °C, 44 °C, and 2-Hz transcutaneous electrical acupoint stimulation (TEAS). The experimental protocol consisted of three phases: baseline, active stimulation, and post-stimulation. All participants received a 40-min stimulation targeting the right PC6 acupoint. EEG data were continuously recorded throughout the experiment.

**Results:**

Microstate analysis revealed that during the stimulation phase, the 2-Hz TEAS group exhibited significantly increased duration, occurrence, and coverage of microstate D compared with the control group, whereas the 40 °C group affected only the duration of microstate D. During the post-stimulation phase, compared with the control group, the 44 °C group showed a decreased duration of microstate A and significantly increased duration and coverage of microstate B, while the 2-Hz TEAS group continued to exhibit sustained effects on the duration of microstate D.

**Conclusion:**

The neuroelectrophysiological signatures of stimulation at PC6 in modulating VRMS are predominantly reflected in the dynamic changes of microstates A, B, and D. Thermal stimulation and low-frequency electrical stimulation engage distinct central modulatory pathways, providing a neuroelectrophysiological basis for optimizing stimulation parameters for in future studies on VRMS.

## Introduction

Recently, virtual reality (VR) technology has been widely applied across multiple domains, including medical rehabilitation, education and training, human–computer interaction, and entertainment (Feng et al., 2025), owing to its high immersion, robust interactivity, and controllable virtual environments (Seong and Park, 2024). Despite providing highly immersive visual experiences, VR frequently induces VR motion sickness (VRMS) (Cohen et al., 2019), with an incidence rate of up to 40% (Wen et al., 2024). VRMS is primarily characterized by dizziness, nausea, vomiting, and spatial disorientation (Kennedy et al., 2010; Rebenitsch and Owen, 2016), which not only compromise user comfort and operational efficiency but also hinder the broader adoption of VR technology in clinical and rehabilitative applications. Current pharmacological management of motion sickness relies predominantly on antihistamines and anticholinergics, which are associated with adverse effects including drowsiness, dry mouth, and blurred vision (Keshavarz and Golding, 2022). Therefore, elucidating the neural mechanisms underlying VRMS and identifying safe and suitable stimulations have become urgent research priorities.

At present, the mechanisms underlying VRMS remain incompletely understood, among which the sensory conflict theory is the most widely accepted (Golding, 1998). This theory proposes that VRMS primarily arises from incongruent information among the visual, vestibular, and somatosensory systems (de Thierry de Faletans et al., 2024). When users are immersed in a virtual environment, the visual system perceives salient motion cues, whereas the vestibular system fails to generate corresponding acceleration signals because the body is not undergoing actual physical movement. This persistent sensory mismatch may ultimately elicit autonomic responses such as dizziness and nausea (Yates, 2009). In addition, several other factors may contribute to the occurrence of VRMS, including postural instability, oculomotor abnormalities, and individual susceptibility (Stanney et al., 2003). VRMS involves not only the peripheral vestibular system but also complex central nervous system processes related to multisensory integration, attentional regulation, autonomic control, and dynamic reconfiguration of brain networks (Yuehua et al., 2025). Given that sensory-conflict-induced alterations in brain function exhibit pronounced temporal dynamics, investigating VRMS from the perspective of dynamic brain network reorganization holds promise for advancing our understanding of its neural underpinnings and identifying effective stimulation targets.

Electroencephalography (EEG), with its high temporal resolution, is a core technique for characterizing dynamic neural activity in the brain (Walla, 2025). It enables real-time capture of millisecond-scale changes in electrophysiological activity during the VR experience (Yang et al., 2023). Previous studies have predominantly employed spectral analysis and event-related potentials to evaluate VRMS-related alterations in brain function. However, these approaches primarily focus on the oscillatory characteristics of localized brain regions and are therefore insufficient to comprehensively capture the temporal dynamics of large-scale brain networks (Wu et al., 2020). Recently, EEG microstate analysis has increasingly emerged as an important method for investigating the temporal dynamics of brain networks (Bai et al., 2024). EEG microstates represent stable, transient, and recurring topographical configurations of whole-brain scalp-recorded electrical activity. Each microstate remains relatively stable for a brief period (80–120 ms) before rapidly transitioning to another stable topographical pattern (Lehmann et al., 1987; Michel and Koenig, 2018). Regarded as the “atoms of thought” (Liu et al., 2024), microstates directly reflect the temporal dynamics of large-scale neural networks (Kleinert et al., 2024). Therefore, EEG microstate analysis offers a novel perspective for investigating VRMS-induced dynamic changes in brain networks.

Classical EEG microstates are typically categorized into four canonical classes: microstates A, B, C, and D. Microstate A is primarily associated with auditory information processing while also contributing, to a lesser extent, to visual information processing. Microstate B is linked to visual mental imagery and visuospatial attention. Microstate C is associated with the detection and evaluation of salient stimuli in both internal and external environments, including the transition from the default mode network to the attentional task network. Microstate D predominantly engages the dorsal attention network (DAN), modulating multisensory integration and goal-directed attentional orienting (Hao et al., 2025). During visually induced VRMS, the durations of microstates A, B, and D are significantly increased, whereas the duration of microstate C is reduced, providing objective evidence of altered dynamic brain activity during VR-induced motion sickness (Nam et al., 2022). EEG microstates therefore provide an appropriate temporal window for observing dynamic network changes during VRMS.

Acupuncture offers a non-pharmacological approach for alleviating VRMS symptoms and has gained widespread recognition for its efficacy in reducing nausea and related symptoms (Liu et al., 2019). Among various acupoints, the Neiguan acupoint (PC6) is commonly used for the treatment of nausea and motion sickness (Frey et al., 2009; Li et al., 2023; Guo et al., 2024). PC6 stimulation can alleviate gastrointestinal responses, such as nausea and vomiting, by modulating the sympathetic-vagal balance, suppressing abnormal gastric myoelectrical activity, and regulating the brain-gut axis (Streitberger et al., 2006; Yu, 2020). Furthermore, both acupuncture and transcutaneous electrical acupoint stimulation (TEAS) at PC6 can effectively reduce chemotherapy-induced and postoperative nausea and vomiting (Yeoh et al., 2016; Wenxi et al., 2024). Although the therapeutic effects of different PC6 stimulation modalities on chemotherapy- and surgery-related nausea and vomiting have been well established, investigations into their efficacy in alleviating VRMS remain limited. In particular, the central regulatory mechanisms through which different peripheral stimulation modalities applied to PC6 modulate VRMS have not yet been systematically investigated.

In the present study, two distinct peripheral stimulation modalities were selected: thermal stimulation (40 °C and 44 °C) and low-frequency TEAS (2-Hz TEAS). A 32 °C condition served as the control. The temperatures of 40 °C and 44 °C represent effective thermal stimulation windows corresponding to mild moxibustion and heat moxibustion, respectively, and these thermal intensities may modulate temperature-sensitive cutaneous pathways and may be associated with biological effects (Wang et al., 2019). A frequency of 2 Hz is a commonly used low-frequency parameter for TEAS in the treatment of nausea and vomiting and has been shown to modulate vagal activity (Cui et al., 2016; Zhang et al., 2025; Zheng et al., 2026). Using a VR-induced visual-vestibular conflict model of motion sickness, this study aimed to compare EEG microstate characteristics across different stimulation conditions, thereby elucidating the central network response profiles and neural mechanisms associated with different PC6 stimulation modalities in modulating VRMS.

## Materials and methods

### Participants

A total of 214 participants were enrolled in this study. Because blinding to the two stimulation modes was not feasible, participants were allocated according to their preference (Wasmann et al., 2019), with 164 in the thermal stimulation group and 50 in the 2 Hz TEAS group. Within the thermal stimulation group, a random sequence generated by the Excel RAND () function was used to further randomly assign participants to a control group (*n* = 55), a 40 °C group (*n* = 54), or a 44 °C group (*n* = 55). In the thermal stimulation group, 7 subjects dropped out due to incompletion, withdrawal, or interruption, and 7 additional subjects were excluded due to poor EEG data quality; all subjects in the 2-Hz TEAS group completed the study. Ultimately, 50 subjects per group were included in the EEG statistical analysis. The flow diagram is shown in Figure 1.

**Figure 1:**
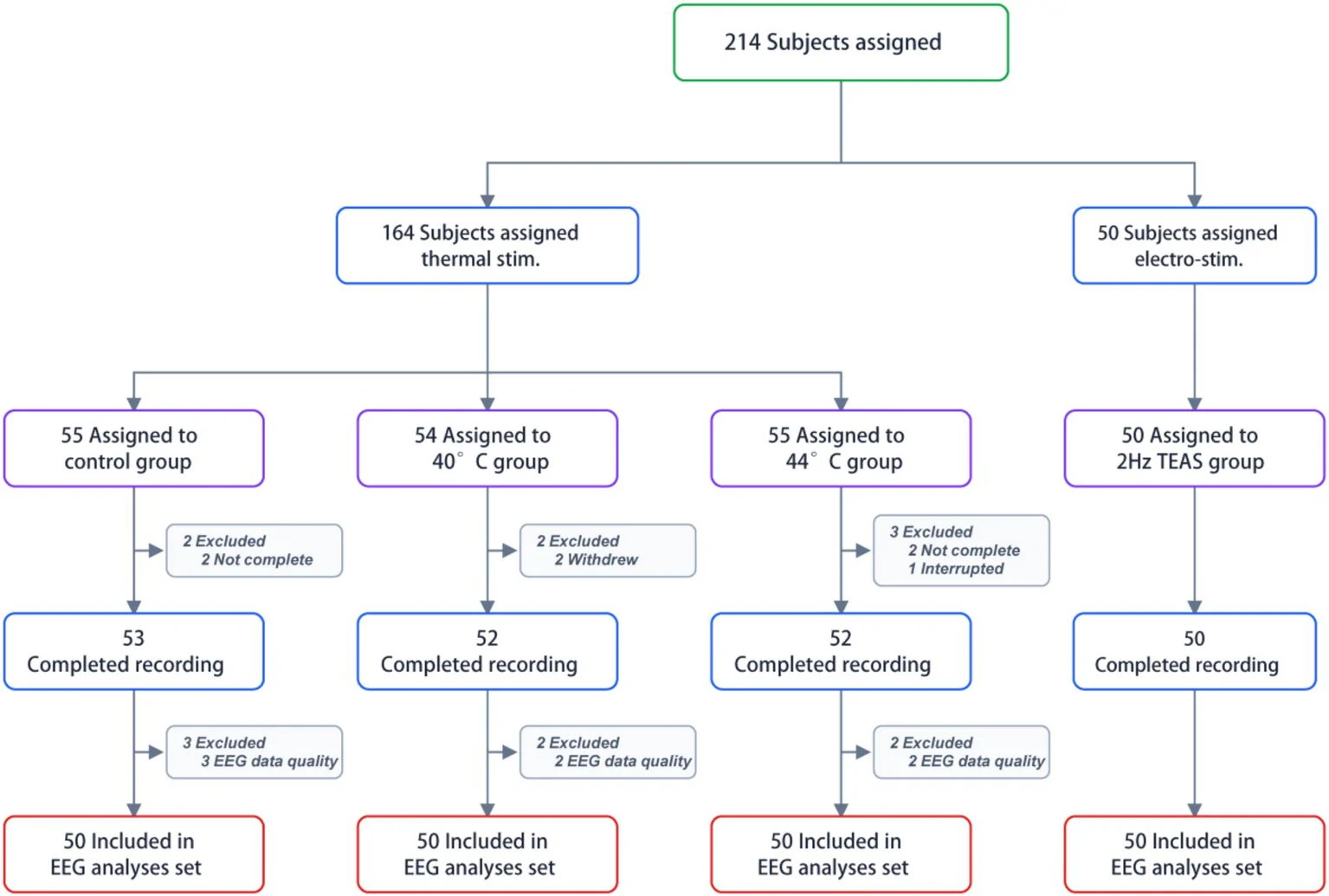
Trial flow diagram.

All participants completed the Golding Motion Sickness Susceptibility Questionnaire (MSSQ) (Golding, 1998). The inclusion criteria were as follows: (1) MSSQ score ≥34; (2) body mass index (BMI) within the normal range (18 ≤ BMI < 25); (3) right-handedness; (4) no use of medications or consumption of caffeine-containing neurostimulatory beverages within the preceding 3 days; and (5) age between 18 and 50 years. The exclusion criteria were as follows: (1) severe psychiatric disorders, including anxiety or depression; (2) a history of gastrointestinal, cardiovascular, visual, or neurosurgical diseases; (3) vestibular disorders, including benign paroxysmal positional vertigo and cervicogenic vertigo; and (4) pregnancy or lactation.

This study was conducted in accordance with the Declaration of Helsinki and approved by the Institutional Ethics Committee (Approval No. LL-2025-11). If a participant experiences acute emesis or severe vertigo during optokinetic drum exposure, the researcher will immediately terminate the exposure, assist the participant into a comfortable position, and provide symptomatic treatment. If necessary, the participant will be evaluated by the study physician and referred to a hospital for further treatment. All adverse events will be documented in detail, and the emergency plans have been reflected in the ethics review. All participants provided written informed consent and received reasonable financial compensation.

### Experimental design

During the experiment, participants remained seated comfortably in a quiet room (Figure 2A). The experimental protocol consisted of three stages. In the first stage (baseline), resting-state EEG was recorded with participants’ eyes open for 16 min, during which they were instructed to fixate on a stationary point on the wall ahead to minimize eye movements. In the second stage, participants wore VR goggles and received VR stimulation while EEG was simultaneously recorded. Concurrently, thermal stimulation or TEAS was applied to the right PC6 acupoint for 40 min. The location of PC6 was determined in accordance with the World Health Organization Standard Acupuncture Point Locations in the Western Pacific Region (Lim, 2010) (Figure 2D). In the third stage, following the stimulation, EEG signals were recorded again under the same conditions as during the baseline phase for 16 min (Figure 2H).

**Figure 2:**
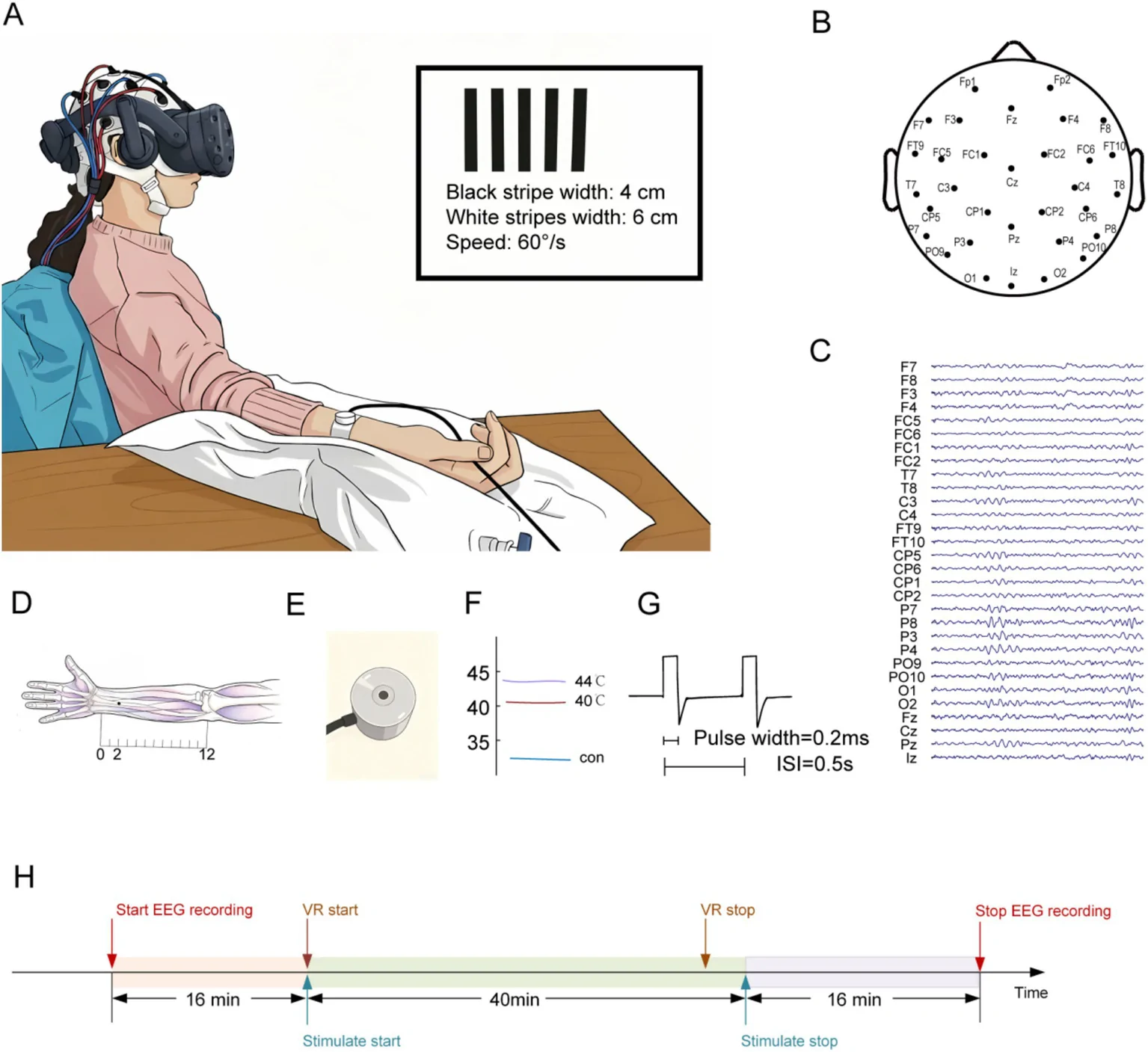
Experimental design. **(A)** Experimental paradigm and VR parameters. **(B)** Electrode placement on the scalp. **(C)** Representative EEG signals from each channel. **(D)** Location of the Neiguan acupoint (PC6). **(E)** EPOS thermode probe. **(F)** Temperature parameters. **(G)** Electrical stimulation parameters. **(H)** Experimental procedure. The experiment consisted of three stages: baseline EEG recording (16 min); stimulation (40 min), during which VR stimulation was delivered concurrently with thermal stimulation (40 °C or 44 °C), 2 Hz-TEAS, or control (32 °C) at PC6; and post-stimulation EEG recording (16 min).

### VR procedure

VR stimulation was delivered using the HTC Vive Pro Eye device (HTC Corporation), which features a head-mounted display with a resolution of 2,880 × 1,600 and operates on the Windows 11 operating system. The professional version of the accompanying handheld controllers was used to control the experimental procedure, and a virtual platform was developed using the Unity engine to generate the visual scene. After donning the VR headset, participants experienced the immersive sensation that their upper body was positioned inside a rotating metal drum. The virtual drum was 282 mm long, 255 mm wide, and 128 mm high. Its inner surface consisted of alternating vertical black and white stripes, with black stripes 4 cm wide and white stripes 6 cm wide. The drum rotated clockwise at a speed of 60°/s (Hu et al., 1991), with a maximum visual angle of 120°. The maximum stimulation duration was 40 min, after which the optokinetic drum stopped rotating immediately. During stimulation, participants could terminate the procedure by pressing a button on the controller if they experienced severe discomfort. No adverse events occurred throughout the study period.

### Stimulation protocols

Thermal stimulation was delivered using the PeriFlux 6,000 EPOS system. The EPOS temperature-controlled probe (PF105-1, Perimed AB, Stockholm, Sweden) was secured to the PC6 acupoint using double-sided tape to deliver stimulation at either 40 °C or 44 °C (Figures 2E,F). TEAS was administered using a Huatuo electronic acupuncture therapeutic apparatus (SDZ-V, Suzhou Medical Supplies Factory Co., Ltd., China). The stimulation waveform was set to continuous wave at a frequency of 2 Hz, with a pulse width of 0.2 ms. The stimulation intensity was set at each participant’s maximum tolerable level and maintained constant throughout the 40-min stimulation period (Figure 2G). The actual stimulation intensity for subjects in the 2-Hz TEAS group is provided in Table S1.

### EEG data acquisition

EEG signals were recorded using the NeurOne EXG amplifier and its accompanying system (Bittium Corporation, Finland), together with a 32-electrode cap (Easycap GmbH, Germany) (Figures 2B,C). Before recording, conductive gel was applied to ensure that electrode impedance was maintained below 5 kΩ. The reference electrode was placed at FCz, and the ground electrode at AFz. Recordings were acquired in DC mode at a sampling rate of 20 kHz. Electrooculography (EOG) was recorded simultaneously, and the level of VR immersion was assessed based on horizontal EOG signals. During EEG acquisition, the VR program automatically embedded different markers into the EEG data at the beginning and end of each phase, thereby synchronizing stimulation and recording to facilitate subsequent EEG segmentation and analysis.

### EEG data preprocessing

EEG data were preprocessed using MATLAB (version R2024b; MathWorks Inc.) in conjunction with the EEGLAB toolbox (Delorme and Makeig, 2004) and custom scripts. The raw EEG signals were band-pass filtered between 1 and 40 Hz by using windowed sinc FIR filter, followed by resampling at 500 Hz (Urigüen and Garcia-Zapirain, 2015). Independent component analysis implemented in EEGLAB was used to remove artifacts, including eye blinks and ocular myoelectric activity (Pascual-Marqui et al., 1995; Jung et al., 2000). Artifacts from head and body movements were visually located and manually rejected. Abnormal channels were identified and interpolated using spherical interpolation. The mean number of interpolated channels were shown in Table S2. Subsequently, the EEG data were referenced to the average. After that, The continuous data were then segmented into 2-s epochs, and epochs containing excessive myogenic activity or movement artifacts were rejected using a threshold of 3 standard deviations. Table S3 reports the amount of usable EEG data retained in each group and phase.

### EEG microstate analysis

Microstate analysis was performed in MATLAB (version R2024b; MathWorks Inc.) using the Microstate EEGlab toolbox (Poulsen et al., 2018). We performed microstate analysis on the cleaned EEG data from the baseline, stimulation and post-stimulation phases, respectively. First, the instantaneous Global Field Power (GFP) was calculated for each participant from processed EEG signals (Figures 3A,B). The spatial configuration of the EEG at the local maximum (peak) of the GFP is considered stable (Wackermann et al., 1993; von Wegner et al., 2017). Therefore, scalp topographies corresponding to GFP peaks were extracted for each participant. The minimum interval between consecutive GFP peaks was set at 10 ms. As the recording durations varied, we extracted scalp topographies at 2000 randomly selected GFP peak locations. GFP peaks that exceeded one times the standard deviation were removed (Liu et al., 2026). Next, a modified K-means clustering algorithm (Maddirala and Veluvolu, 2022) was applied to group highly similar topographies into the same microstate class (i.e., template map). An optimized segmentation scheme with 1,000 iterations was employed. As the four template maps (microstates A, B, C, and D) have been the most common and highly reproducible in previous resting-state EEG studies (Zappasodi et al., 2017; Michel and Koenig, 2018; Pan et al., 2020; Kim et al., 2021; Liu et al., 2021; Liu et al., 2026), we have selected 4 as the optimal number of clusters (Figure 3C). The four template maps were subsequently back-fitted to all the EEG data, thereby transforming the EEG signals into microstate time series (Figure 3D). Finally, temporal smoothing was applied to the microstate sequences, and segments with durations < 20 ms were reassigned to the next best fitting template map. We reported the variance in EEG activity explained by all template maps (global explained variance, GEV). The temporal parameters of each participant in the four template maps were calculated, including duration, time coverage, and occurrence (Khanna et al., 2014; Khanna et al., 2015; Haydock et al., 2025). The duration refers to the average duration of each microstate (in milliseconds). The time coverage refers to the proportion of time taken up by each microstate in the entire microstate sequence (as a percentage). The occurrence refers to the frequency with which each microstate occurs in a second (counts per second).

**Figure 3:**
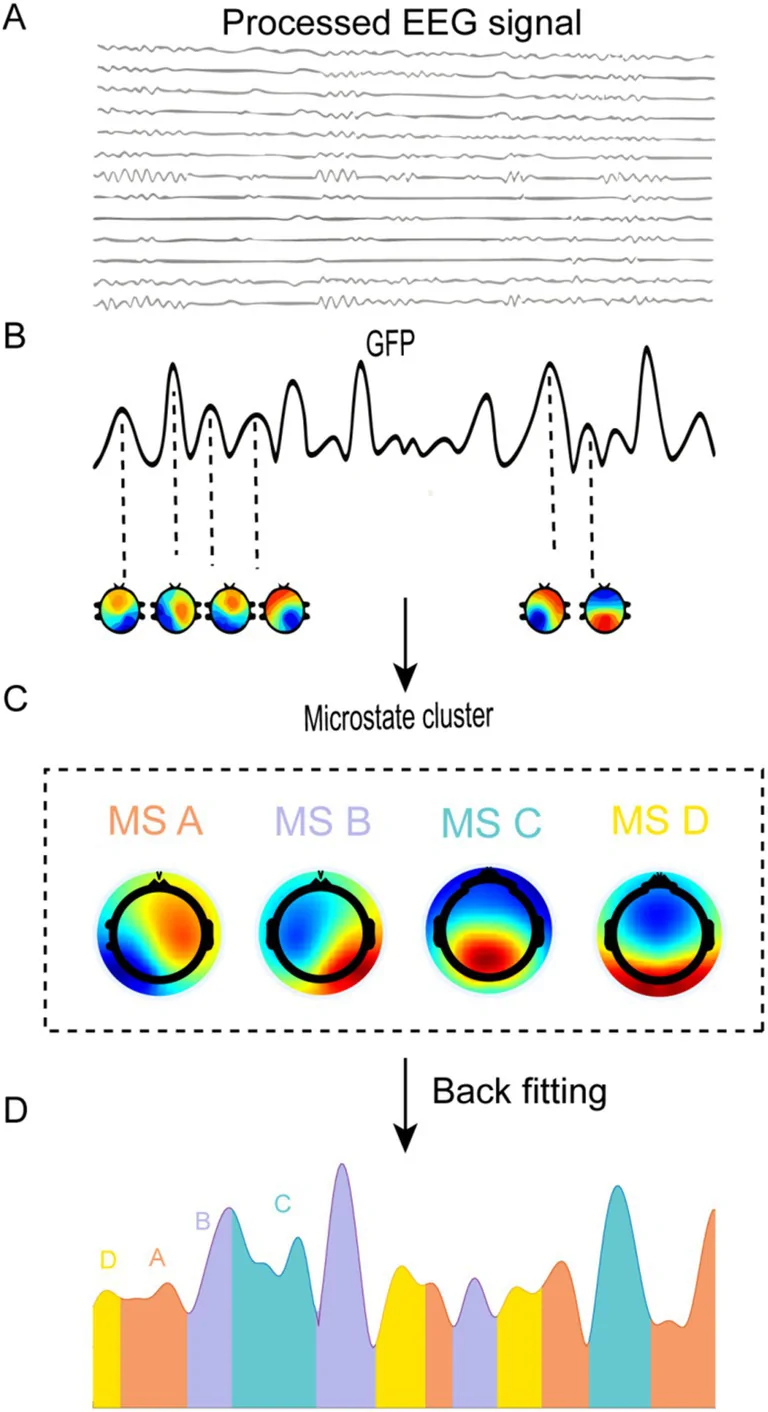
Microstate analysis. **(A)** Preprocessed EEG data. **(B)** Global field power (GFP) was computed, and GFP peak topographies with optimal signal-to-noise ratio and topographical stability were extracted as clustering samples. **(C)** An improved k-means clustering algorithm was applied to classify the topographies into four canonical microstate classes based on spatial correlation. **(D)** A back-fitting procedure was then performed to match the microstate templates to the scalp topographies at each time point, thereby transforming the EEG data into microstate time series.

### Statistical analyses

All statistical analyses were performed using IBM SPSS Statistics (version 27.0). First, baseline demographic characteristics were compared across groups to assess between-group balance. Categorical variables were analyzed using the chi-square test. When the expected frequencies did not satisfy the assumptions of the chi-square test, Fisher’s exact test was used instead. The normality of continuous variables, including age, height, and body weight, was assessed using the Shapiro–Wilk test. Normally distributed variables were compared among groups using one-way analysis of variance, whereas non-normally distributed variables were analyzed using the Kruskal–Wallis *H* test.

This study focused on the differences between each experimental group and the control group. Given the between-group imbalance at baseline, EEG microstate analyses were performed using baseline-adjusted linear regression models. The assumption of homogeneity of regression slopes was first evaluated (*α* = 0.05). If the assumption was satisfied, baseline values were entered into the model as continuous covariates. If the assumption was violated, a group-by-baseline interaction term was included to account for heterogeneous regression slopes and ensure unbiased estimation of treatment effects. Variance inflation factors for all independent variables indicated no evidence of substantial multicollinearity, supporting the robustness of the regression models.

When the assumption of homogeneity of regression slopes was met, results were reported as baseline-adjusted regression coefficients (B), corresponding 95% confidence intervals (CIs), and two-sided *p* values. When the assumption was violated, adjusted means and corresponding *p* values were reported. We used a MATLAB script to perform false discovery rate (FDR) correction on the *p*-value clusters in the statistical analysis of microstate parameters. All relevant data are available in Tables S4 and S5.

To explore the temporal dynamics of microstate characteristics and their association with the subjective symptoms of VR motion sickness, we performed principal component analysis (PCA) on the changes (i.e., differences) in three metrics (duration, occurrence, and coverage) of the four microstate classes relative to the baseline, and extracted the first principal component (PC1), which explained the largest proportion of variance (Wu et al., 2025), as a composite index of the global dynamic characteristics of microstates. Spearman’s rank correlation analysis was applied to assess the associations between PC1 and VR exposure duration, as well as subjective nausea ratings, in the four groups and the total sample. Correlation coefficients were denoted as *ρ*, and all *p* values were adjusted using the false discovery rate (FDR) method, with statistical significance defined as *P_*FDR < 0.05. Since VR exposure duration bears practical relevance only within the stimulation period and nausea ratings reflect subjective VRMS symptom changes, we restricted correlation analyses to stimulation-phase data.

Additionally, Kaplan–Meier survival curves and adjusted survival curves for the exposure duration of the four groups were generated, and between-group differences were compared using the Log-rank test. Meanwhile, to exclude the potential confounding effect of sex on the significant results, we performed sex-stratified subgroup sensitivity analyses to assess the interaction between sex and group.

## Results

### Subjects characteristics

Detailed demographic characteristics of all participants are presented in Table 1. No significant differences were observed among the groups in terms of sex (*p* = 0.147), height (*p* = 0.611), weight (*p* = 0.648), or age (*p* = 0.959), MSSQ score (Kruskal–Wallis *H* rank-sum test, *p* = 0.007), or VR exposure time (Kruskal–Wallis *H* rank-sum test, *p* = 0.511). For the MSSQ motion sickness score with inter-group imbalance, a univariate Cox regression analysis was further conducted. The results showed that it had no significant association with the risk of the outcome event (HR = 1.004, 95% CI: 0.971–1.039, *p* = 0.797), and it met the proportional hazards assumption. Therefore, it was not included in the adjusted Cox regression model.

**Table 1:** Subject characteristics.

Characteristics	32 °C (*N* = 50)	40 °C (*N* = 50)	44 °C (*N* = 50)	2 Hz (*N* = 50)	*p-*value
Sex					0.147
Male	15 (30)	13 (26)	10 (20)	6 (12)	
Female	35 (70)	37 (74)	40 (80)	44 (88)	
Height (cm)	165.50 (160.75–170.25)	163.50 (159.75–174.00)	163.00 (160.00–168.00)	165.00 (160.00–170.00)	0.611
Weight (kg)	60.00 (55.00–66.00)	59.00 (53.00–70.00)	58.50 (52.38–65.50)	57.50 (52.00–64.00)	0.648
Age (years)	23.50 (22.00–25.00)	23.50 (23.00–24.00)	23.00 (22.00–25.00)	23.00 (22.00–25.00)	0.959
MSSQ score	113.78 (67.12–145.43)	105.51 (70.37–146.03)	116.33 (67.36–159.39)	70.99 (53.96–113.18)	0.007
VR exposure time	36.00 (16.75–40.00)	40.00 (17.75–40.00)	31.50 (19.00–40.00)	40.00 (22.00–40.00)	0.511

Continuous variables are presented as medians (interquartile ranges). Categorical variables are presented as *n* (%).

### Cluster evaluation

In the baseline EEG data, the mean GEV across the four prototypical microstates for all participants was 71.55% (SEM = 0.24%). By group, the GEVs for the 32 °C, 40 °C, 44 °C, and 2-Hz TEAS groups were 71.72% (SEM = 0.48%), 71.03% (SEM = 0.46%), 71.93% (SEM = 0.59%), and 71.54% (SEM = 0.38%), respectively.

In the stimulation EEG data, the mean GEV across the four prototypical microstates for all participants was 73.48% (SEM = 0.28%). By group, the GEVs for the 32 °C, 40 °C, 44 °C, and 2-Hz TEAS groups were 73.23% (SEM = 0.51%), 73.51% (SEM = 0.55%), 73.68% (SEM = 0.60%), and 73.50% (SEM = 0.57%), respectively.

In the post-stimulation EEG data, the mean GEV across the four prototypical microstates for all participants was 72.44% (SEM = 0.23%). By group, the GEVs for the 32 °C, 40 °C, 44 °C, and 2-Hz TEAS groups were 72.09% (SEM = 0.41%), 72.28% (SEM = 0.46%), 72.95% (SEM = 0.50%), and 72.43% (SEM = 0.46%), respectively.

### Microstate analysis results

The four microstates (A–D) identified in this study (Figure 4A, 5A). During the stimulation phase, no significant differences were observed among the groups in the duration, occurrence, or coverage of microstate A (Figures 4B–D), B (Figures 4E–G), or C (Figures 4H–J) (all *p* > 0.05). The effects of 2-Hz TEAS were predominantly observed in microstate D. Compared with the control group, 2-Hz TEAS significantly increased the duration (adjusted mean: 69.857 vs. 64.683, *p* = 0.044, *P*_FDR = 0.044) (Figure 4K), occurrence (*B* = 0.235, 95% CI: 0.003–0.467, *p* = 0.047, *P*_FDR = 0.047) (Figure 4L), and coverage (*B* = 0.042, 95% CI: 0.010–0.074, *p* = 0.012, *P*_FDR = 0.012) (Figure 4M) of microstate D. In addition, the 40 °C stimulation also significantly increased the duration of microstate D (adjusted mean: 69.370 vs. 64.683, *p* = 0.017, *P*_FDR = 0.017) (Figure 4K).

**Figure 4:**
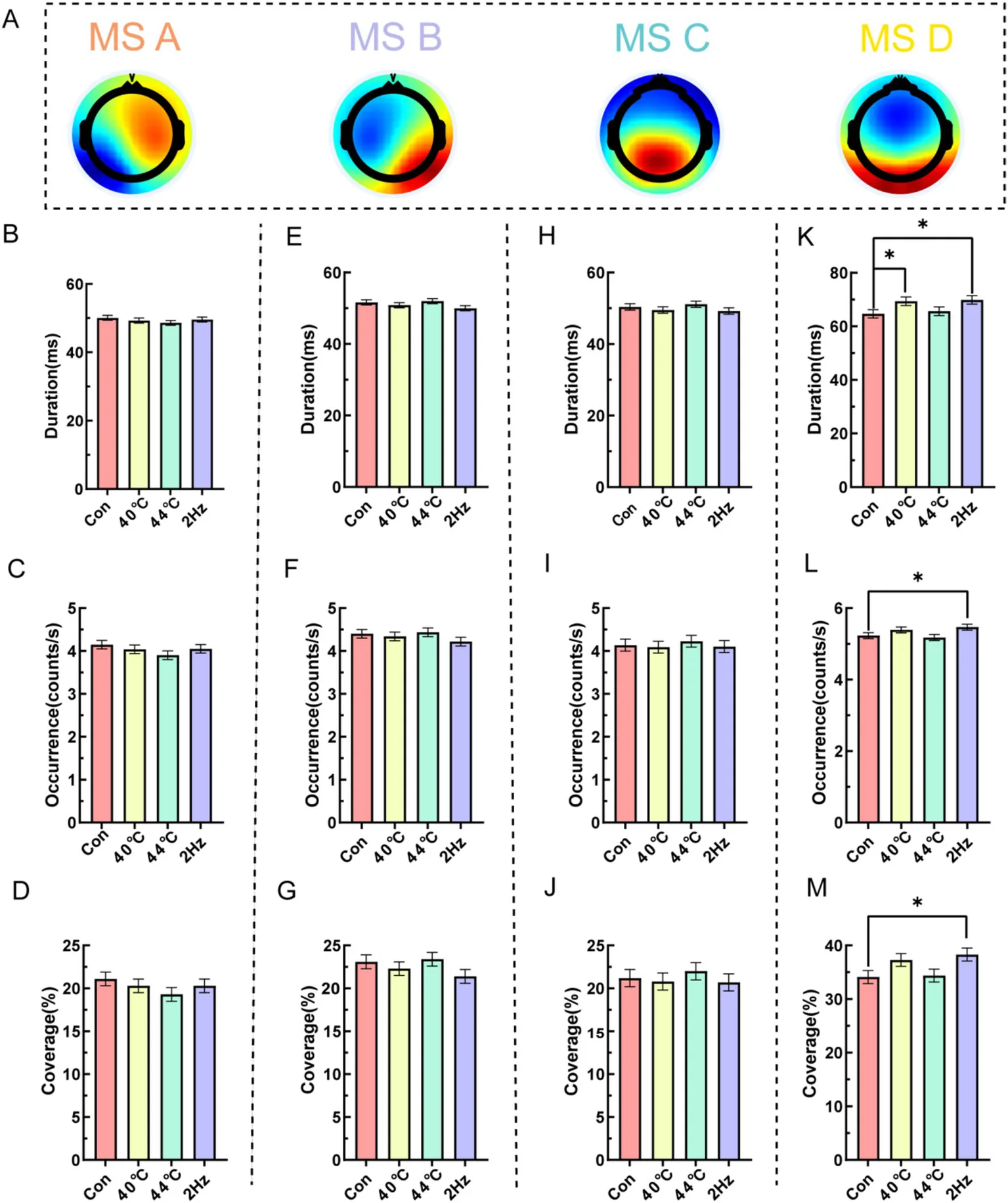
Adjusted means and standard errors of EEG microstate parameters during the stimulation phase. **(A)** The four microstate classes. Panels **(B–D)**, **(E–G)**, **(H–J)**, and **(K–M)** show the duration, occurrence, and coverage of microstates A, B, C, and D, respectively. *p < 0.05.

During the post-stimulation phase, the effects of 44 °C stimulation became apparent. Compared with the control group, 44 °C stimulation significantly decreased the duration of microstate A (adjusted mean: 47.998 vs. 49.086, *p* = 0.017, *P*_FDR = 0.017) (Figure 5B), while no significant changes were observed in its occurrence or coverage (Figure 5C,D).Meanwhile, the duration of microstate B was significantly increased (*B* = 2.202, 95% CI: 0.128–4.276, *p* = 0.038, *P*_FDR = 0.038) (Figure 5E) whereas its occurrence showed no significant difference (Figure 5F). In addition, the coverage of microstate B was significantly increased (*B* = 0.020, 95% CI: 0.001–0.040, *p* = 0.042, *P*_FDR = 0.042) (Figure 5G). The 2-Hz TEAS group also maintained the effects observed during the stimulation phase. Compared with the control group, the duration of microstate D remained significantly increased (adjusted mean: 64.078 vs. 61.078, *p* = 0.011, *P*_FDR = 0.011) (Figure 5K), while its occurrence and coverage showed no significant differences (Figure 5L,M).

**Figure 5:**
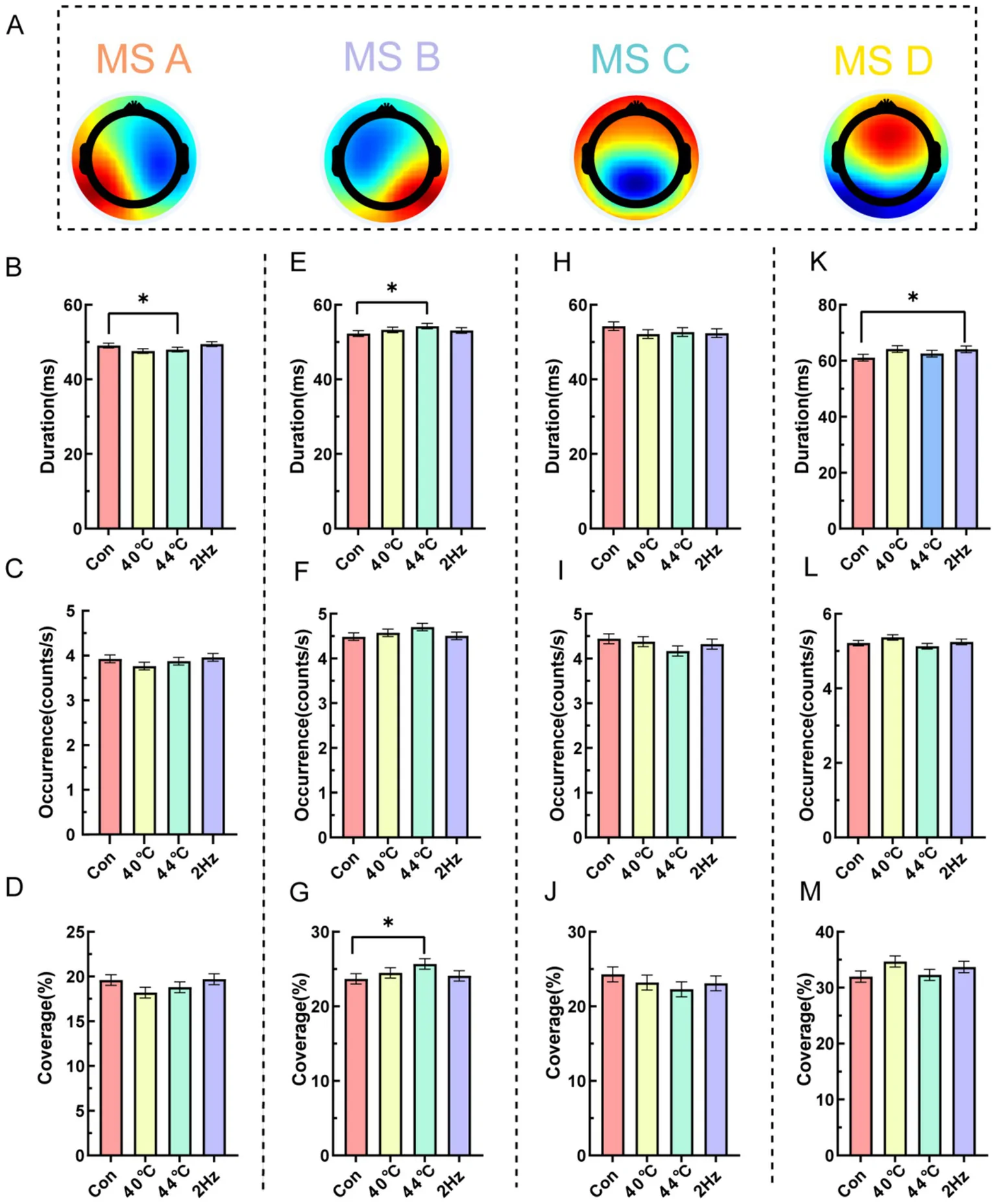
Adjusted means and standard errors of EEG microstate parameters during the post-stimulation phase. **(A)** The four microstate classes. Panels **(B–D)**, **(E–G)**, **(H–J)**, and **(K–M)** show the duration, occurrence, and coverage of microstates A, B, C, and D, respectively. *p < 0.05.

None of the stimulation modalities exerted significant effects on microstate C during either the stimulation or post-stimulation phase (Figures 4H–J,5H–J).

### Correlation analysis results

Spearman’s rank correlation analyses revealed no significant correlations between PC1 and VR exposure duration during the stimulation phase in individual group. However, in the overall sample, PC1 was significantly positively correlated with VR exposure duration (*ρ* = 0.203, *p* = 0.004, *P*_FDR = 0.020) (Table 2). No significant correlation was observed between PC1 and subjective motion sickness scores in either the individual groups or the overall sample (all *P*_FDR > 0.05) (Table 3).

**Table 2:** Spearman’s correlation between PC1 and VR exposure time.

Group	*n*	*ρ*	*P*	*P*_FDR
32 °C	50	−0.0539	0.710	0.710
40 °C	50	0.301	0.0337	0.0561
44 °C	50	0.204	0.155	0.194
2-Hz TEAS	50	0.307	0.0302	0.0561
Overall	200	0.203	0.004	0.020

**Table 3:** Spearman’s correlation between PC1 and subjective motion sickness score.

Group	*n*	*ρ*	*P*	*P*_FDR
32 °C	50	−0.114	0.431	0.719
40 °C	50	−0.0425	0.769	0.769
44 °C	50	−0.0677	0.640	0.769
2-Hz TEAS	50	−0.166	0.250	0.624
Overall	200	−0.0944	0.184	0.624

### Survival analysis and sensitivity analysis results

Survival analysis of intervention exposure time across the four groups is presented in Supplementary Figure S1. No significant difference was observed among the four groups. Subgroup sensitivity analysis stratified by sex showed no significant interaction between sex and group in all phases (all *P* int. > 0.05; Supplementary Figures S2, S3). More detailed accounts of sex subgroup sensitivity analyses are provided in the Supplementary material.

## Discussion

Previous studies have demonstrated that stimulation of PC6 can alleviate symptoms such as dizziness and nausea (Lee et al., 2013; Yılmaz et al., 2023). The present study used EEG microstate analysis to investigate the effects of different PC6 stimulation modalities on VRMS by comparing EEG microstate characteristics following thermal stimulation and 2-Hz TEAS across different experimental stages. The findings suggest that, during the stimulation phase, microstate D is involved in the regulatory effects of 2-Hz TEAS, whereas during the post-stimulation phase, microstates A and B are involved in the regulatory effects of 44 °C thermal stimulation. In both the stimulation and post-stimulation phases, microstate C was not involved in the regulatory effects of PC6 stimulation.

Microstate D is closely associated with the DAN and reflects goal-directed attention to external sensory information and cognitive regulation (Kim, 2014). Previous studies have shown that microstate D primarily involves brain regions such as the bilateral middle frontal gyrus, intraparietal sulcus, and inferior parietal lobule, which play critical roles in attentional control and visual-vestibular multisensory integration (Custo et al., 2017). In a study on VR cybersickness, Nam et al. (2022) observed prolonged duration of microstate D. They attributed this increase to persistent visual-vestibular conflict, indicating enhanced activation of the attentional network. Therefore, an increase in microstate D during VR exposure should not not be simply interpreted as a therapeutic or beneficial neural response, but may instead reflect brain state changes in response to sensory conflict and cognitive demand. In the present study, 2-Hz TEAS increased the duration, occurrence, and coverage of microstate D. This finding may be related to the effects of TEAS on attentional processing and sensory information processing networks (Sajid et al., 2026). Previous evidence has also demonstrated that PC6 stimulation strengthens functional activity within the frontoparietal network (Yu et al., 2016). Moreover, low-frequency TEAS modulates brain regions involved in emotional and attentional processing, among which the inferior parietal lobule serves as a key node of the attention network (Lopes Alves et al., 2025). The immersive VR environment induces typical visual-vestibular sensory conflict, while the painful sensation produced by 2-Hz TEAS may further amplify the sensory discrepancy, increase arousal, and enhance attentional orienting toward external stimuli (Cong et al., 2024), and may also affect network activity through somatosensory input (Jousmäki, 2000). Accordingly, changes in microstate D may reflect brain state changes shaped by attention, arousal, somatosensory input, or sensory conflict. However, the relationship between microstate D changes and VRMS symptom changes remains to be further investigated.

Microstate A primarily involves brain regions such as the bilateral superior temporal gyrus, middle temporal gyrus, and left insula and is associated with the auditory network and global vigilance-arousal level of the brain (Bai et al., 2025). Microstate B mainly involves the bilateral occipital cortex and posterior cingulate gyrus and is associated with visual imagery, including self-visualization and autobiographical memory (Tarailis et al., 2024). Previous studies have shown that thermal stimulation, as an effective somatosensory input, can modulate brain neural activity (Lv et al., 2017). In the post-stimulation phase of the present study, the decreased duration of microstate A and the increased duration of microstate B in the 44 °C group may reflect a redistribution of neural resources between external environmental vigilance and internal visual information processing. After the termination of immersive VR visual input and localized thermal stimulation, the overall level of vigilance toward the external environment and somatic sensations declines, allowing the brain to shift its processing resources toward memory retrieval and internal processing of residual VR experiences.

Microstate C is primarily associated with the salience and default mode networks (Deodato et al., 2024). Previous studies have indicated that alterations in microstate C are associated with conditions such as Alzheimer’s disease, anxiety, and depression. In patients with Alzheimer’s disease and mild cognitive impairment, the occurrence and coverage of microstate C are significantly reduced (Piton et al., 2026), whereas in patients with schizophrenia and depression, the coverage of microstate C is abnormally increased (Lin et al., 2025). These findings suggest that changes in microstate C parameters may primarily reflect brain network dysfunction under pathological conditions. In the present study, however, no significant changes in microstate C parameters were observed, possibly because the participants were healthy volunteers with brain functional networks in a normal physiological state.

Different stimulation modalities exhibited distinct time-effect trajectories, representing the most notable finding of the present study. The effects of 2-Hz TEAS followed a dual regulatory pattern characterized by immediate onset and sustained maintenance, with its effects consistently targeting the attention-related microstate D. The effects of 40 °C thermal stimulation were observed only during the stimulation phase, representing a transient regulation of immediate onset but rapid decay, also targeting microstate D. In contrast, the effects of 44 °C thermal stimulation emerged exclusively during the post-stimulation phase, exhibiting a delayed-onset pattern with its effects shifting toward microstates A and B, which are associated with visual and auditory processing. Moreover, 2-Hz TEAS has been proposed to be involved in the recruitment of thick myelinated Aβ fibers and thinly myelinated Aδ fibers, which conduct signals to the central nervous system, and its effects may be sustained through synaptic plasticity changes (He et al., 2025). Although 40 °C thermal stimulation can modulate central neural activity during stimulation, the intensity of its afferent input may be insufficient to induce long-lasting synaptic plasticity; consequently, its effects diminish after stimulation ceases. The delayed effects observed following 44 °C thermal stimulation may involve transient receptor potential vanilloid 1 (TRPV1) related pathways (Bang et al., 2023). TRPV1 is a well-characterized noxious heat-sensitive channel, with an activation threshold generally reported to be around 43 °C (Tominaga and Iwata, 2025). Therefore, 44 °C thermal stimulation may induce TRPV1-related sensory pathways and subsequently influence central neural network activity (Laing and Dhaka, 2016). TRPV1 activation may trigger asynchronous glutamate release at central synapses of primary afferents, and this enhancement can persist for more than 20 min after repeated activation, suggesting that thermal stimulation may produce delayed effects through sustained synaptic gain (Peters et al., 2010). Our previous studies showed that local blood flow returned linearly after stimulation below 40 °C, whereas the blood flow changes induced by 44 °C stimulation lasted substantially longer (Wang et al., 2018), possibly reflecting sustained activation of the nitric oxide-dependent system. This persistent peripheral modulation may provide input signals for delayed responses at the central level. Studies have shown that cold and heat stimulation enhanced cortical excitability 30 min after the end of stimulation (Magara et al., 2021), suggesting that the expression of thermal stimulation effects may be delayed. Since the present study only employed scalp EEG microstate analysis, which primarily reflects large-scale cortical network dynamics, it cannot directly determine the specific types of fibers recruited, TRPV1-mediated mechanisms, or synaptic plasticity changes. Therefore, interpretations involving Aβ/Aδ fiber recruitment, synaptic plasticity, and TRPV1-mediated mechanisms should be considered hypotheses requiring further validation in future studies.

Furthermore, the present correlation analysis indicated that PC1, which was mainly composed of microstate features, increased with VR exposure duration in the overall sample, suggesting that sustained visual-vestibular conflict may progressively alter brain activity. This finding is consistent with previous evidence that VR exposure disturbs EEG microstate temporal dynamics (Nam et al., 2022). However, PC1 was not significantly correlated with subjective nausea scores, may be incomplete synchrony between neural activity changes and the experience of subjective symptoms (Napadow et al., 2013).

Although all stimulation modalities targeted the same acupoint, PC6, they engaged distinct brain networks and exhibited different temporal response profiles. This phenomenon of “same acupoint, different effects” challenges the traditional understanding of the relationship between acupoints and their therapeutic effects, suggesting that the central responses elicited by acupoint stimulation may be dependent on the mode of stimulation. These findings provide a novel perspective on the neural mechanisms underlying acupoint stimulation and may help guide the clinical selection of stimulation parameters for acupoint-based stimulations.

## Limitations

The present study has several limitations. First, EEG recordings were uniformly acquired under an eyes-open paradigm, without a corresponding eyes-closed condition, making it impossible to compare the characteristics of the four microstate parameters between the two resting states. Second, the present study focused solely on PC6 and did not examine the influence of the same stimulation mode at other acupoints or sites. Future studies could include non-acupoint or sham stimulation conditions to verify acupoint specificity and further clarify the underlying neural mechanisms. Third, the functional network interpretation of microstates in this study was mainly based on scalp surface topography, without formal cortical source localization modeling such as eLORETA. Future studies could combine eLORETA or other source localization methods to further verify the cortical sources of microstates and their corresponding functional networks. Future studies should refine the experimental design to address these limitations.

## Conclusion

During the stimulation phase, 2-Hz TEAS primarily associated with changes in microstate D, whereas 44 °C thermal stimulation predominantly associated with changes in microstates A and B during the post-stimulation phase. The study provides objective neurophysiological evidence for understanding the central effects of non-invasive acupoint-based stimulation in VRMS.

## Data Availability

The original contributions presented in the study are included in the article/Supplementary material, further inquiries can be directed to the corresponding authors.
