# Supplementary material for: Different modes of stimulation at the Neiguan acupoint (PC6) regulate different EEG microstates under VR immersion conditions
Source: Front Comput Neurosci. 2026 Oct 22;20:1938329. doi: 10.3389/fncom.2026.1938329 (PMC13736752; doi:10.3389/fncom.2026.1938329)
Supplement: Supplementary file 1 [file Data_Sheet_1.PDF]

Supplementary Materials for

Different modes of stimulation at the Neiguan acupoint (PC6) regulate different EEG microstates under VR immersion conditions

The file includes:
Table S1-S5
Figure S1-S3

Table S1

sub	Stimulus intensity
1	20
2	30
3	30
4	28
5	25
6	26
7	35
8	40
9	35
10	35
11	32
12	36
13	23
14	40
15	33
16	35
17	33
18	40
19	37
20	37
21	38
22	35
23	38
24	38
25	27
26	31
27	22
28	35
29	37
30	24
31	35

32	25
33	25
34	41
35	35
36	40
37	32
38	28
39	50
40	40
41	35
42	28
43	29
44	35
45	32
46	26
47	29
48	28
49	23
50	40

Table S2. Number of bad channels.

**Phase**	**32 °C (*n*=50)**	**40 °C (*n*=50)**	**44 °C (*n*=50)**	**2 Hz (*n*=50)**
Baseline	1.32	1.12	1.3	1.34
Stimulation	1.36	1.56	1.36	1.8
Post-stimulation	1.36	1.64	1.48	1.56

Values are means.

Table S3. The amount of usable EEG data retained (s).

**Phase**	**32 °C (*n*=50)**	**40 °C (*n*=50)**	**44 °C (*n*=50)**	**2 Hz (*n*=50)**
Baseline	605.08	571.12	571.2	477.52
Stimulation	760.04	773.72	848.48	744.8
Post-stimulation	494	464.76	469.6	413.36

Values are means.

Table S4. Adjusted data during the stimulation phase.

MS	variable	contrast	Ajusted Mean±SE	coefficients(95% CI)	P values	P_FDR
MSA	Duration	Con vs40℃	50.088±0.722vs 49.286±0.721	-0.803(-2.814,1.208)	0.432	
		Con vs44℃	50.088±0.722 vs 48.625 ± 0.721	-1.464(-3.478,0.551)	0.154	
		Con vs2Hz	50.088±0.722 vs 49.599 ± 0.723	-0.490(-2.510,1.531)	0.633	
	Occurrence	Con vs40℃	4.147±0.100 vs 4.038 ±0.100	-0.110(-0.390,0.170)	0.441	
		Con vs44℃	4.147±0.100 vs 3.902±0.100	-0.246(-0.525,0.034)	0.085	
		Con vs2Hz	4.147±0.100 vs 4.052 ±0.100	-0.095(-0.375,0.185)	0.503	
	Coverage	Con vs40℃	0.211±0.008vs 0.203 ± 0.008	-0.008(-0.029,0.013)	0.461	
		Con vs44℃	0.211 ± 0.008 vs 0.193 ± 0.008	-0.018(-0.040,0.003)	0.090	
		Con vs2Hz	0.211 ± 0.008 vs 0.203 ± 0.008	-0.007(-0.029,0.014)	0.491	
MSB	Duration	Con vs40℃	51.643±0.728 vs 50.862±0.719	-0.781(-2.804,1.241)	0.447	
		Con vs44℃	51.643±0.728 vs 51.969 ± 0.719	0.326(-1.698,2.349)	0.751	
		Con vs2Hz	51.643±0.728 vs 49.995 ± 0.721	-1.648(-3.682,0.387)	0.112	
	Occurrence	Con vs40℃	4.401 ± 0.099 vs 4.340 ± 0.099	-0.061(-0.338,0.216)	0.663	
		Con vs44℃	4.401 ± 0.099 vs 4.435 ± 0.099	0.034(-0.243,0.311)	0.808	
		Con vs2Hz	4.401 ± 0.099 vs 4.219 ± 0.099	-0.182(-0.460,0.095)	0.197	
	Coverage	Con vs40℃	0.231 ± 0.008 vs 0.223 ± 0.008	-0.008(-0.030,0.014)	0.487	
		Con vs44℃	0.231 ± 0.008 vs 0.234 ± 0.008	0.003(-0.019,0.025)	0.808	
		Con vs2Hz	0.231 ± 0.008 vs 0.214 ± 0.008	-0.017(-0.039,0.005)	0.135	
MSC	Duration	Con vs40℃	50.383 ± 0.897 vs 49.521 ± 0.896	-0.862(-3.371,1.647)	0.499	
		Con vs44℃	50.383 ± 0.897 vs 51.116 ± 0.901	0.733(-1.758,3.224)	0.562	

		Con vs2Hz	50.383 ± 0.897 vs 49.211 ± 0.902	-1.172(-3.696,1.353)	0.361	
	Occurrence	Con vs40℃	4.137 ± 0.138 vs 4.090 ± 0.137	-0.047(-0.434,0.340)	0.811	
		Con vs44℃	4.137 ± 0.138 vs 4.228 ± 0.138	0.091(-0.290,0.471)	0.638	
		Con vs2Hz	4.137 ± 0.138 vs 4.104 ± 0.139	-0.032(-0.423,0.359)	0.871	
	Coverage	Con vs40℃	0.212 ± 0.010 vs 0.208 ± 0.010	-0.004(-0.032, 0.023)	0.762	
		Con vs44℃	0.212 ± 0.010 vs 0.220 ± 0.010	0.008(-0.019, 0.035)	0.562	
		Con vs2Hz	0.212 ± 0.010 vs 0.207 ± 0.010	-0.005(-0.033, 0.022)	0.706	
MSD	Duration	Con vs40℃	64.683 ± 1.571 vs 69.370 ± 1.607	-37.65(-68.44, -6.86)	0.017[a]	0.017
		Con vs44℃	64.683 ± 1.571 vs 65.639 ± 1.603	-12.68(-41.77, 16.41)	0.391[a]	
		Con vs2Hz	64.683 ± 1.571 vs 69.857 ± 1.578	-32.32(-63.71, -0.94)	0.044[a]	0.044
	Occurrence	Con vs40℃	5.230 ± 0.084 vs 5.390 ± 0.083	0.160(-0.071, 0.392)	0.174	
		Con vs44℃	5.230 ± 0.084 vs 5.177 ± 0.084	-0.053(-0.289, 0.183)	0.658	
		Con vs2Hz	5.230 ± 0.084 vs 5.465 ± 0.083	0.235 (0.003, 0.467)	0.047	0.047
	Coverage	Con vs40℃	0.341 ± 0.012 vs 0.373 ± 0.012	-0.032(-0.001, 0.064)	0.057	
		Con vs44℃	0.341 ± 0.012 vs 0.344 ± 0.012	0.003(-0.029, 0.036)	0.842	
		Con vs2Hz	0.341 ± 0.012 vs 0.383 ± 0.012	0.042 (0.010, 0.074)	0.012	0.012

Values are adjusted means ± SE and adjusted coefficients (95% CI). All comparisons are vs.Con. [a], Significant group-by-baseline interaction; *P* values are raw.

Table S5. Adjusted data during the post-stimulation phase.

MS	variable	contrast	Ajusted Mean ± SE	95%CI	P values	P_FDR
MSA	Duration	Con vs40℃	49.086 ± 0.64 vs 47.58 ± 0.63	-4.90(-20.64, 10.84)	0.544[a]	
		Con vs44℃	49.086 ± 0.64 vs 47.998 ± 0.63	19.23(3.79, 34.66)	0.017[a]	0.017
		Con vs2Hz	49.086 ± 0.64 vs 49.48 ± 0.64	7.34(-9.48, 24.17)	0.403[a]	

	Occurrence	Con vs40℃	3.923 ± 0.086 vs 3.762 ± 0.086	-0.161(-0.400, 0.078)	0.186	
		Con vs44℃	3.923 ± 0.086 vs 3.871 ± 0.086	-0.053(-0.292, 0.186)	0.664	
		Con vs2Hz	3.923 ± 0.086 vs 3.956 ± 0.086	0.033 (-0.207, 0.272)	0.789	
	Coverage	Con vs40℃	0.196 ± 0.006 vs 0.182 ± 0.006	-0.015(-0.033, 0.003)	0.102	
		Con vs44℃	0.196 ± 0.006 vs 0.188 ± 0.006	-0.009(-0.026, 0.009)	0.345	
		Con vs2Hz	0.196 ± 0.006 vs 0.197 ± 0.006	0.001 (-0.017, 0.018)	0.955	
MSB	Duration	Con vs40℃	52.298 ± 0.765 vs53.27± 0.738	1.110 (-0.963, 3.183)	0.292	
		Con vs44℃	52.298 ± 0.765 vs54.29± 0.739	2.202 (0.128, 4.276)	0.038	0.038
		Con vs2Hz	52.298 ± 0.765 vs53.10± 0.755	1.055 (-1.030, 3.140)	0.320	
	Occurrence	Con vs40℃	4.487 ± 0.084 vs4.573± 0.083	0.085 (-0.148, 0.318)	0.471	
		Con vs44℃	4.487 ± 0.084 vs4.702± 0.083	0.214 (-0.019, 0.448)	0.072	
		Con vs2Hz	4.487 ± 0.084 vs4.507± 0.083	0.020 (-0.214, 0.254)	0.865	
	Coverage	Con vs40℃	0.237 ± 0.007 vs 0.245 ± 0.007	0.008 (-0.011, 0.028)	0.417	
		Con vs44℃	0.237 ± 0.007 vs 0.257 ± 0.007	0.020 (0.001, 0.040)	0.042	0.042
		Con vs2Hz	0.237 ± 0.007 vs 0.241 ± 0.007	0.004 (-0.016, 0.024)	0.690	
MSC	Duration	Con vs40℃	54.276 ± 1.169 vs52.140±1.168	-2.136(-5.407, 1.135)	0.199	
		Con vs44℃	54.276 ± 1.169 vs52.697±1.174	-1.579(-4.827, 1.668)	0.339	
		Con vs2Hz	54.276 ± 1.169 vs52.386±1.176	-1.890(-5.181, 1.401)	0.259	
	Occurrence	Con vs40℃	4.442±0.111vs 4.379±0.111	-0.063(-0.375, 0.250)	0.692	
		Con vs44℃	4.442 ± 0.111 vs4.169 ± 0.11	-0.273(-0.579, 0.034)	0.081	
		Con vs2Hz	4.442 ± 0.111 vs4.324± 0.112	-0.118(-0.434, 0.197)	0.460	
	Coverage	Con vs40℃	0.243 ± 0.010 vs 0.232 ± 0.010	-0.011(-0.038, 0.017)	0.439	

		Con vs44℃	0.243 ± 0.010 vs 0.223 ± 0.010	-0.020(-0.047, 0.007)	0.148	
		Con vs2Hz	0.243 ± 0.010 vs 0.231 ± 0.010	-0.012(-0.039, 0.016)	0.393	
MSD	Duration	Con vs40℃	61.078 ± 1.185 vs64.13± 1.212	-10.26(-33.48, 12.97)	0.385[a]	
		Con vs44℃	61.078 ± 1.185 vs 62.562 ± 1.209	3.13(-18.82, 25.07)	0.779[a]	
		Con vs2Hz	61.078 ± 1.185 vs64.07± 1.191	-30.98(-54.66, -7.31)	0.011[a]	0.011
	Occurrence	Con vs40℃	5.215 ± 0.074 vs 5.366 ± 0.074	0.151 (-0.055, 0.356)	0.149	
		Con vs44℃	5.215 ± 0.074 vs5.131± 0.074	-0.084(-0.294, 0.125)	0.427	
		Con vs2Hz	5.215 ± 0.074 vs 5.248 ± 0.074	0.033(-0.173, 0.239)	0.752	
	Coverage	Con vs40℃	0.320 ± 0.010 vs 0.347 ± 0.010	0.027(0.000, 0.054)	0.051	
		Con vs44℃	0.320 ± 0.010 vs 0.323 ± 0.010	0.003 (-0.024, 0.030)	0.827	
		Con vs 2Hz	0.320 ± 0.010 vs 0.337 ± 0.010	0.017 (-0.010, 0.044)	0.215	

Values are adjusted means ± SE and adjusted coefficients (95% CI). All comparisons are vs.Con. [a], Significant group-by-baseline interaction; *P* values are raw.

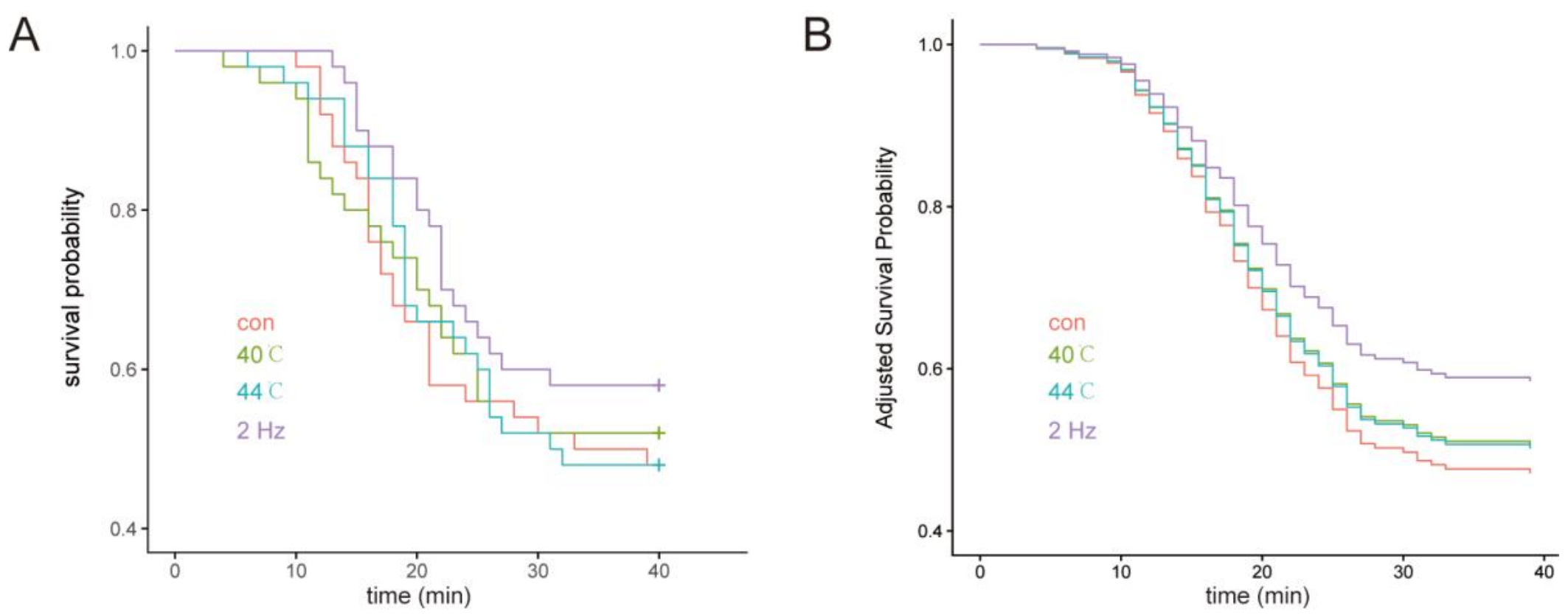

Fig. S1 Survival Curves of different groups over Time.(A) unadjusted and (B) adjusted survival curves across groups over time.The red, green, blue, and purple lines represent the con (32℃), 40℃, 44℃, and 2 Hz groups, respectively. *P* > 0.05.

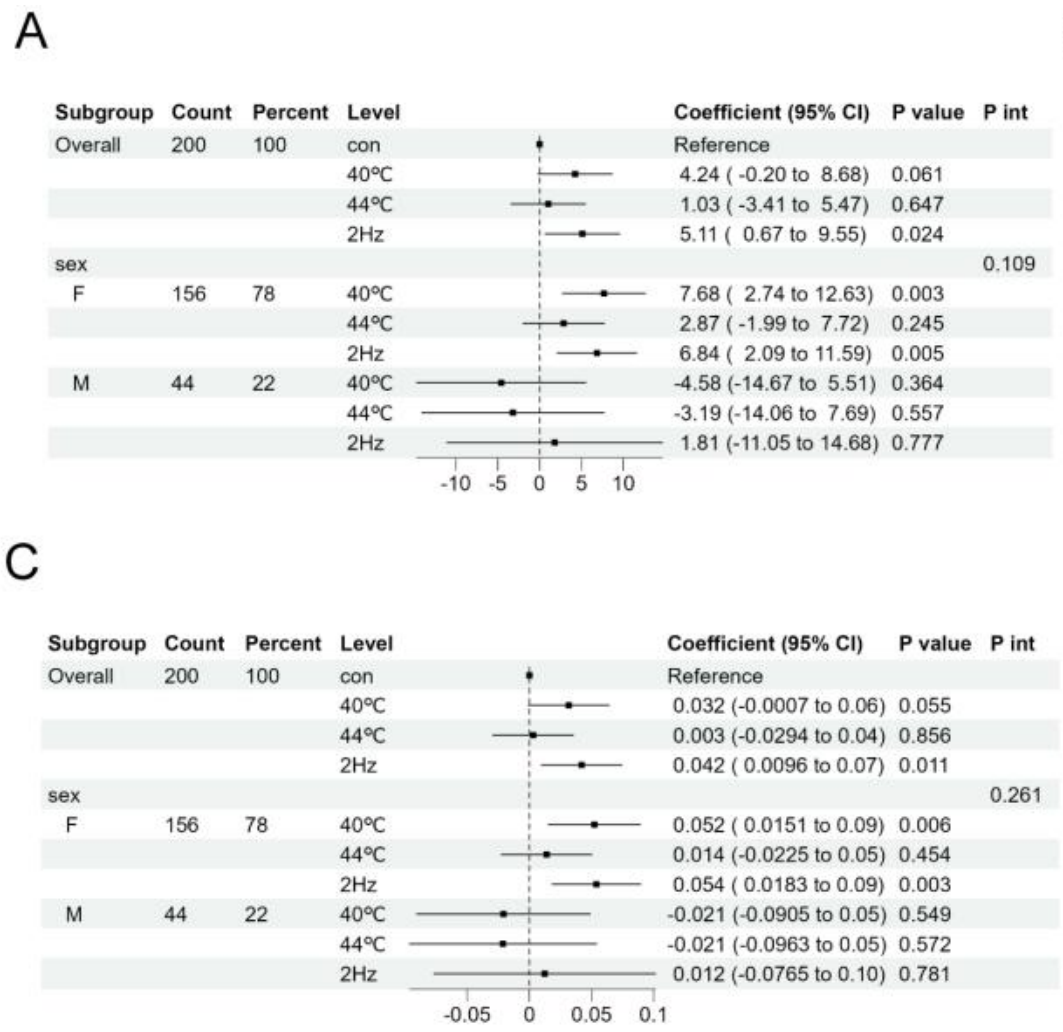

Fig. S2 Sensitivity analysis for the interaction between group and sex during the stimulation phase.(A) Duration, (B) occurrence, and (C) coverage of microstate D.Abbreviations: con, control; CI, confidence interval; P int, P value for interaction.

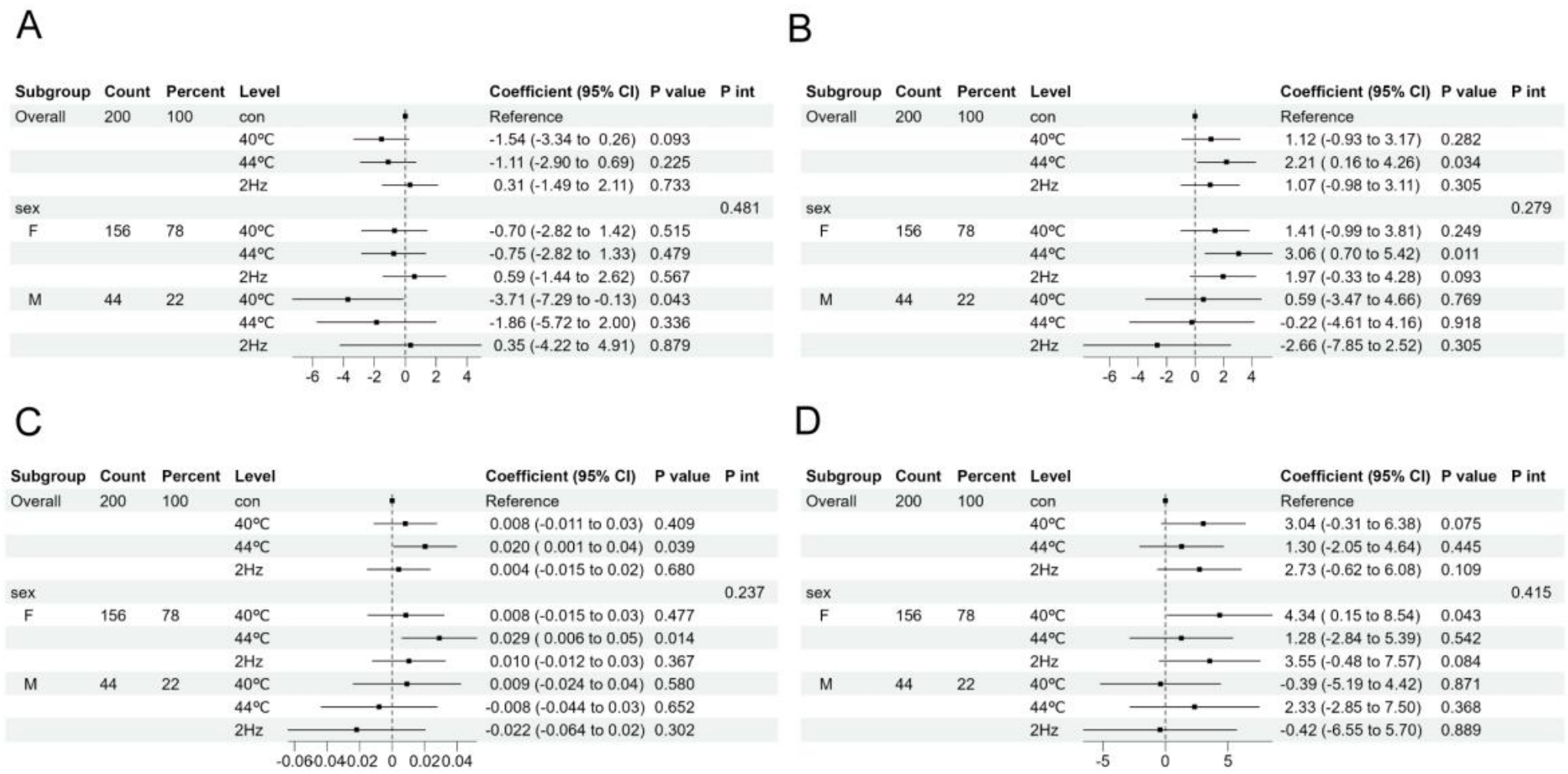

Fig. S3 Sensitivity analysis for the interaction between group and sex during the post-stimulation phase.(A) duration of microstate A; (B) duration and (C) coverage of microstate B; (D) duration of microstate D.Abbreviations:con, control; CI, confidence interval; P int, P value for interaction.
